# A Randomized Study of Lubiprostone for Opioid-Induced Constipation in Patients with Chronic Noncancer Pain

**DOI:** 10.1111/pme.12437

**Published:** 2014-04-09

**Authors:** Byron Cryer, Seymour Katz, Ricardo Vallejo, Anca Popescu, Ryuji Ueno

**Affiliations:** * Department of Internal Medicine, Digestive and Liver Diseases, University of Texas Southwestern Medical SchoolDallas, Texas, USA; † Gastroenterology and Hepatology, Veterans Affairs North Texas Health Care SystemDallas, Texas, USA; ‡ Long Island Clinical Research AssociatesGreat Neck, New York, USA; § Millennium Pain CenterBloomington, Illinois, USA; ¶ Department of Neurology, Thomas Jefferson University HospitalPhiladelphia, Pennsylvania, USA; ** Sucampo Pharma Americas, LLCBethesda, Maryland, USA; †† Sucampo AGZug, Switzerland

**Keywords:** Bowel Movement, ClC-2, Opioid, Opioid-Induced Constipation, Placebo-Controlled Trial, Prostone

## Abstract

**Objective:**

To evaluate the efficacy and safety of oral lubiprostone for relieving symptoms of opioid-induced constipation (OIC) in patients with chronic noncancer pain.

**Design:**

Prospective, randomized, double-blind, placebo-controlled trial.

**Setting:**

Seventy-nine US and Canadian centers.

**Subjects:**

Patients aged ≥18 years with OIC, defined as <3 spontaneous bowel movements (SBMs) per week.

**Methods:**

Patients received lubiprostone 24 mcg or placebo twice daily for 12 weeks. The primary endpoint was change from baseline in SBM frequency at week 8.

**Results:**

Among randomized patients (N = 418; lubiprostone, N = 210; placebo, N = 208), most completed the study (lubiprostone, 67.1%; placebo, 69.7%). The safety and efficacy (intent-to-treat) populations included 414 (lubiprostone, N = 208; placebo, N = 206) and 413 (lubiprostone, N = 209; placebo, N = 204) patients, respectively. The mean (standard deviation) age was 50.4 (10.9) years; most patients were female (64.4%) and white (77.7%). Changes from baseline in SBM frequency rates were significantly higher at week 8 (*P* = 0.005) and overall (*P* = 0.004) in patients treated with lubiprostone compared with placebo. Pairwise comparisons showed significantly greater overall improvement for abdominal discomfort (*P* = 0.047), straining (*P* < 0.001), constipation severity (*P* = 0.007), and stool consistency (*P* < 0.001) with lubiprostone compared with placebo. Moreover, patients rated the effectiveness of lubiprostone as significantly (*P* < 0.05) better than placebo for 11 of 12 weeks. The most common treatment-related adverse events (AEs) with lubiprostone and placebo were nausea (16.8% vs 5.8%, respectively), diarrhea (9.6% vs 2.9%), and abdominal distention (8.2% vs 2.4%). No lubiprostone-related serious AEs occurred.

**Conclusion:**

Lubiprostone effectively relieved OIC and associated signs and symptoms and was well tolerated in patients with chronic noncancer pain (http://clinicaltrials.gov/ct2/show/NCT00595946).

## Introduction

Chronic pain affects approximately 25–44% of adults in the United States and Europe 1–3. In the United States alone, it is estimated that 100 million adults have chronic pain, resulting in direct and indirect costs of $560–635 billion [4]. Although opioid therapy is central to management of moderate-to-severe noncancer pain [5], it is limited by manifestations of opioid-induced constipation (OIC), including straining, hard stools, incomplete evacuation, and abdominal bloating [6]. Binding of opioids to μ-opioid receptors on enteric neurons promotes increased absorption of fluid and electrolytes, such as chloride, leading to reduced gastrointestinal motility and the resultant clinical symptoms of OIC [7]. A systematic review estimated the prevalence of OIC at 41% among patients with chronic noncancer pain [8]. Importantly, patients with OIC frequently report that severe constipation imposes an additional quality-of-life burden in addition to their chronic pain [9]. Despite the consequences of uncontrolled OIC, until recently, no medications have been approved by regulatory authorities to treat this condition in patients with chronic noncancer pain. Although over-the-counter options (e.g., stool softeners, bulk laxatives, and enemas) are available, well-controlled data on their safety and efficacy for the treatment of OIC are lacking. Thus, there is a need for therapies with proven safety and efficacy to improve control of OIC.

Lubiprostone was approved in April 2013 by the US Food and Drug Administration (FDA) for the treatment of OIC in adults with chronic noncancer pain at a dose of 24 mcg twice daily (BID), in addition to its previous indications for adults with chronic idiopathic constipation (CIC) at a dose of 24 mcg BID and for adult women with irritable bowel syndrome with constipation (IBS-C) at a dose of 8 mcg BID [10]. Lubiprostone bypasses enteric neurons by locally activating ClC-2 chloride channels in the apical membrane of epithelial cells in the gastrointestinal tract, thus promoting intestinal secretion of chloride, and consequently fluid, which helps with softening stools and facilitating bowel movements (BMs), without affecting serum electrolyte concentrations [11]. In rodent models, lubiprostone reversed morphine-induced suppression of chloride secretion [12] and accelerated ileocecal transit time without affecting opioid-induced analgesia [13]. This mechanism of action suggests how lubiprostone may improve the signs and symptoms that are associated with OIC. The objectives of the current study were to assess the efficacy and safety of lubiprostone 24 mcg BID for the treatment of OIC in adult patients with chronic noncancer pain.

## Methods

### Patients

Men and nonpregnant, nonlactating women aged ≥18 years were eligible for enrollment if treated for chronic noncancer pain with a stable dose of any full-agonist opioid for ≥30 days before screening and were to continue utilizing a consistent opioid regimen (i.e., <30% change) throughout the study. Women of childbearing potential were required to use effective contraception. Patients were required to have OIC, defined as a mean of <3 spontaneous bowel movements (SBMs) (i.e., BMs without the use of laxatives or stool softeners within the past 24 hours) per week during the screening period, as well as 1 or more of the following characteristics associated with ≥25% of SBMs during each screening week: hard or very hard stools, sensation of incomplete evacuation, or moderate to very severe straining. Patients were required to discontinue medications that affect gastrointestinal motility (e.g., anticholinergics, laxatives, stool softeners, and tricyclic antidepressants) or are known to relieve or cause constipation, bloating, or constipation-related symptoms from the screening visit through study completion. Patients on a stable fiber supplement for ≥30 days before screening were allowed to continue the same regimen.

Patients were excluded because of opioid therapy for cancer-related pain, abdominal pain, scleroderma, or drug addiction; mechanical bowel obstruction (e.g., tumor, hernia, or obstructive polyps); organic disorder of the large or small bowel (e.g., ulcerative colitis or Crohn's disease); constipation unrelated to opioids (e.g., due to dietary issues such as malnutrition); or congenital or endocrine disorders (e.g., hypothyroidism or diabetes). Patients were also excluded because of unexplained loss of >5% of body mass or gastrointestinal or abdominal surgery within 90 days before screening; bowel resection at any time; impaired renal function; clinically significant cardiovascular disease; or clinically significant liver or lung disease, psychiatric or neurologic disorders (e.g., spinal cord disorders), or other systemic disease, unless the investigator felt that these conditions would neither interfere with study procedures nor pose an increased risk to the patient. Patients who participated in another clinical study in the past 30 days or previously received lubiprostone were excluded.

### Study Design and Treatment

This randomized, double-blind, placebo-controlled, phase III study (ClinicalTrials.gov entry NCT00595946) was conducted at gastroenterology, pain, and general practice clinics (76 in the United States and three in Canada) between 8/2007 and 3/2009. The protocol and study procedures were approved by an institutional review board at each center and conducted according to the Declaration of Helsinki, International Conference on Harmonisation Guidelines for Good Clinical Practice, and federal and local regulations. All study participants provided written informed consent before the initiation of study procedures. The authors had access to the study data and reviewed and approved the final manuscript.

After a 3-week screening period, eligible patients were randomized 1:1 at baseline (week 0) to self-administered oral lubiprostone 24 mcg BID (48 mcg daily) or matching placebo capsules. Treatment was assigned using an interactive voice or web response system according to a computer-generated randomization table generated by an independent statistician and stratified by study center; patients, investigators, and study staff were blinded to assignments. One capsule of study treatment was to be taken with food and 8 oz of water at each morning and evening meal for 12 weeks. Patients returned to the clinic at weeks 4, 8, 12, and 14 and were contacted by telephone at weeks 1, 6, and 10 to review electronic diaries, report new adverse events (AEs), follow up on existing AEs, note any changes in concomitant medications, and assess compliance with study treatment. Physical examinations and collection of samples for clinical laboratory tests were conducted at all postbaseline clinic visits. Vital signs were measured, and pregnancy tests were performed at all clinic visits. A 12-lead electrocardiogram measurement was obtained at the screening visit and week 14.

A permanent change to once-daily dosing was permitted at the investigator's discretion after consultation with the medical monitor if a patient experienced an AE (severe nausea, severe diarrhea, or other event) for ≥3 consecutive days. In patients with no BM within a 3-day period, the investigator was permitted to prescribe a 10 mg bisacodyl suppository. If this rescue medication was ineffective, the investigator was permitted to recommend an enema; if the enema failed, immediate short-term use of another treatment, except polyethylene glycol 3350 or tegaserod, was permitted. The use of rescue medication was permitted during screening and the entire treatment period, except for ≤24 hours before administration of the first dose of study medication or during the first week of treatment.

### Efficacy Assessments

The study objectives were to evaluate the efficacy and safety of lubiprostone for the treatment of OIC in adult patients with chronic noncancer pain. The primary endpoint was change from baseline (i.e., last 2 weeks of screening) in the frequency of SBMs at week 8, calculated based on the patient's daily record of BMs and rescue medication use. An SBM was defined as any BM that did not occur within 24 hours after rescue medication use.

Secondary endpoints included changes from baseline in the frequency of SBMs at week 12 and overall (average of all diary entries), percentage of patients with the first postdose SBM within 24 and 48 hours of administering study medication, and overall responder rates. An overall responder was defined as a patient who was in the study for at least 8 weeks, achieved at least a moderate response (≥3 SBMs) for at least 50% of the study weeks, did not use rescue medication during the response week, and did not withdraw from the study due to lack of efficacy. Secondary endpoints also included patient-assessed overall treatment effectiveness and overall mean change from baseline in constipation-associated symptoms, bowel habits, and stool consistency. Straining associated with SBMs, abdominal bloating, abdominal discomfort, and constipation severity was scored on a 5-point scale ranging from 0 (absent) to 4 (very severe). Stool consistency was assessed using a 5-point scale ranging from 0 (very loose) to 4 (very hard). Bowel habit regularity was assessed based on a 7-point scale ranging from 1 (very regular) to 7 (very irregular). Patient subjective impressions of overall treatment effectiveness were assessed on a 5-point scale of 0 (not at all effective) to 4 (extremely effective).

### Safety Assessments

AEs from the first dose of study medication until 7 days after the last dose (i.e., treatment-emergent AEs) were recorded and classified by investigators according to Medical Dictionary for Regulatory Activities (MedDRA 13.1) preferred terms. Serious AEs (SAEs) were those that resulted in death, a life-threatening event (risk of death), hospital admission, prolongation of hospitalization, persistent or significant disability/incapacity, or congenital anomaly/birth defect, or were an important medical event (judged serious by the investigator). Patients performed self-assessments of nausea using the modified Functional Living Index-Emesis (MFLIE) questionnaire (nausea subscale; range, 9–63) and pain using the Brief Pain Inventory-short form (BPI-SF; range 0–10).

### Statistical Analysis

A sample size of 130 patients per group was estimated to provide ≥93% power to detect a change from baseline in weekly SBM frequency with lubiprostone that was greater than the change with placebo by a mean (standard variation [SD]) value of 1.5 (3.5) at week 8. To accommodate withdrawals and dose reductions, an initial sample of 210 patients per group was planned in order to have 130 patients per group at week 8. Inferential tests for analysis of efficacy were two-tailed at a significance level of α = 0.05.

Demographics and baseline disease characteristics were summarized for all randomized patients. Randomized patients who received ≥1 dose of double-blind study medication and provided ≥1 treatment-period diary entry were defined as the intent-to-treat (ITT) population for analysis of efficacy. The safety analysis group included all randomized patients who took ≥1 dose of double-blind study medication.

The primary endpoint, change from baseline in SBM frequency at week 8, was analyzed for patients who did not have a dose reduction before week 8, using the van Elteren test stratified by pooled study center; changes from baseline SBM frequency at week 12 and overall were analyzed similarly. The Kaplan–Meier estimates of median time to first SBM were compared using a Cox regression model. The proportion of patients with their first SBM within 24 and 48 hours after the first dose of study medication was compared between treatment groups using a χ^2^ test. Overall mean changes from baseline in constipation-associated symptoms and treatment effectiveness were compared between groups using the van Elteren test stratified by pooled study center. Although no missing data were imputed for the primary endpoint, where appropriate, secondary efficacy variables were analyzed using the last observation carried forward.

Demographic data, baseline disease status, and safety data were summarized using descriptive statistics and, where appropriate, compared using a χ^2^ or Fisher's exact test for categorical variables and a *t* test, Wilcoxon rank sum test, or analysis of variance for continuous variables.

## Results

### Patients

After meeting eligibility requirements, 418 patients were randomized to lubiprostone 24 mcg BID (N = 210) or placebo (N = 208; Supporting Information Fig. S1). One patient assigned to receive lubiprostone was inadvertently given placebo; per protocol, this patient was included in the lubiprostone group for efficacy analyses but in the placebo group for safety and other analyses. Within the lubiprostone arm, 208 and 209 patients were evaluated for safety and efficacy (ITT population), respectively, whereas in the placebo arm, 206 and 204 patients were included in the safety and ITT populations, respectively. Completion rates were similar: 67.1% (141/210) in the lubiprostone group and 69.7% (145/208) in the placebo group (*P* = 0.573). Withdrawals due to AEs were more frequent in the lubiprostone group than in the placebo group (16/210 [7.6%] vs 6/208 [2.9%], respectively), a difference that reached statistical significance (*P* = 0.046; Fisher's exact test). During double-blind treatment, patients in each arm received the same mean (SD) total daily dose of study medication (1.7 [0.3] capsules per day; *P* = 0.687; Wilcoxon rank sum test) and had a similar mean (SD) treatment duration (67.2 [28.9] and 71.6 [25.8] days in the lubiprostone and placebo arms, respectively; *P* = 0.090; Wilcoxon rank sum test).

Demographic and baseline disease characteristics were generally well balanced between the treatment arms; the mean morphine equivalent daily dose was significantly higher in the lubiprostone arm compared with the placebo arm (Table 1). The mean age was 50.4 years, and approximately two-thirds of patients were women. Most patients were white (77.7%), 18.7% were black, 1.7% were Asian, 1.0% were American Indian or Alaska Native, and 1.0% were of other races. Patients were taking relatively large doses of opioids (most commonly oxycodone, hydrocodone, and morphine) and were experiencing constipation-related symptoms, such as hard stools, that were generally moderate to severe in intensity.

**Table 1:** Patient demographics and disease status at baseline for all randomized patients

Demographics of All Randomized Patients	Placebo BID (N = 208)	Lubiprostone 24 mcg BID (N = 210)	*P* value*
Mean ± SD age, y	50.3 ± 12.0	50.5 ± 9.7	0.975
Sex, N (%)			0.541
Women	137 (65.9)	132 (62.9)	
Men	71 (34.1)	78 (37.1)	
Race, N (%)†			0.821
White	164 (79.2)	160 (76.2)	
Black or African American	36 (17.4)	42 (20.0)	
Asian	4 (1.9)	3 (1.4)	
American Indian or Alaska Native	2 (1.0)	2 (1.0)	
Other	1 (0.5)	3 (1.4)	
Disease status, mean ± SD			
Number of SBMs per week‡	1.5 ± 1.0	1.4 ± 1.1	0.793
Consistency of SBMs§∥	3.0 ± 0.8	3.0 ± 0.9	0.555
Constipation severity¶,#	2.3 ± 0.8	2.3 ± 0.8	0.951
Straining associated with SBMs§^,^#	2.6 ± 0.8	2.7 ± 0.9	0.431
Abdominal discomfort¶^,^#	2.1 ± 0.7	2.1 ± 0.7	0.980
Abdominal bloating¶^,^#	2.2 ± 0.8	2.2 ± 0.8	0.879
Bowel habit regularity‡,**	4.7 ± 1.6	4.5 ± 1.7	0.382
Modified Functional Living Index-Emesis¶^,^††	46.0 ± 13.0	46.4 ± 12.7	0.734
Brief Pain Inventory short form – Pain Severity‡‡^,^§§	4.4 ± 3.0	5.0 ± 2.8	0.106
Morphine equivalents,∥∥ mg/d	237 ± 451	265 ± 407	0.012
Rescue medication usage,¶ %	15.4 ± 19.0	13.9 ± 18.6	0.550

BID = twice daily; SBM = spontaneous bowel movement.

* *P* values for continuous variables are from a van Elteren test stratified by pooled site; *P* values for categorical variables are from Fisher's exact test.

† Placebo, N = 207; lubiprostone, N = 210.

‡ Placebo, N = 204; lubiprostone, N = 209.

§ Placebo, N = 187; lubiprostone, N = 176.

∥ 5-point scale: 0 = very loose; 1 = loose; 2 = normal; 3 = hard; 4 = very hard.

¶ Placebo, N = 205; lubiprostone, N = 209.

# 5-point scale: 0 = absent; 1 = mild; 2 = moderate; 3 = severe; 4 = very severe.

** 7-point scale: 1 = very regular; 7 = very irregular.

†† Subscale: 9–63.

‡‡ Placebo, N = 189; lubiprostone, N = 191.

§§ Scale: 0–10.

∥∥ Placebo, N = 204; lubiprostone, N = 208.

### SBMs

**Figure 1:**
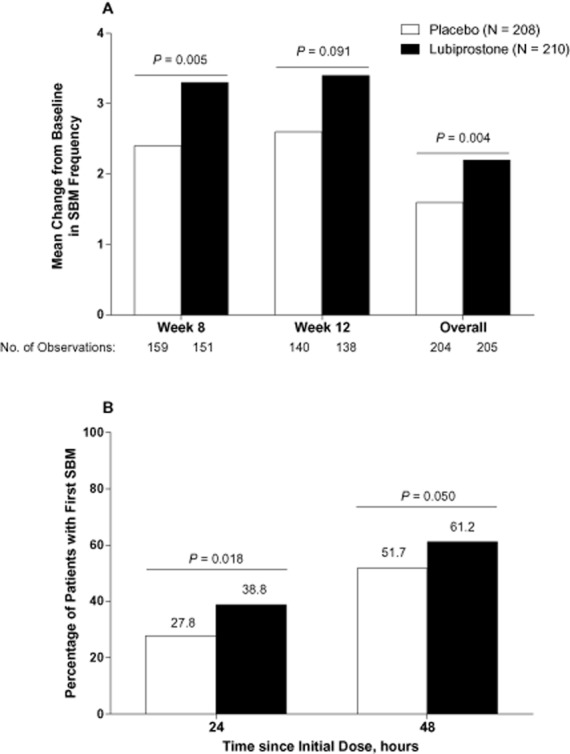
(A) Change from baseline in frequency of spontaneous bowel movements (SBMs) in patients treated with lubiprostone 24 mcg twice daily compared with placebo (intent-to-treat population). *P* values from van Elteren tests stratified by pooled center. (B) Percentage of patients with first SBM within 24 and 48 hours of initial dose of lubiprostone 24 mcg twice daily compared with placebo. *P* values are from χ^2^ tests. No imputation of missing data was performed.

The change from baseline in SBM frequency was significantly greater with lubiprostone compared with placebo at week 8 for patients in the ITT population who did not have a dose reduction by week 8 (the prespecified primary endpoint; mean, 3.3 vs 2.4 SBMs/week, *P* = 0.005; median 2.9 vs 1.4 SBMs/week; Figure 1A). A further analysis of the primary endpoint to include patients regardless of dose reduction by week 8 yielded comparable findings (mean, 3.3 vs 2.4 SBMs/week, *P* = 0.004). The overall change from baseline in SBM frequency was also significantly greater with lubiprostone compared with placebo (mean, 2.2 vs 1.6 SBMs/week, *P* = 0.004); at week 12, the same pattern was observed but did not achieve significance (Figure 1A). This was likely due to the relatively fewer number of patients remaining in the study at week 12. A significantly greater percentage of patients treated with lubiprostone compared with placebo achieved their first SBM within 24 (*P* = 0.018) and 48 (*P* = 0.050) hours after administration of the first dose of study medication (Figure 1B). Although the median time to first SBM in patients treated with lubiprostone was reduced by almost half compared with that of placebo (28.5 vs 46.0 hours, respectively), the difference between treatment groups did not reach statistical significance (*P* = 0.053).

### Constipation-Related Signs and Symptoms

**Figure 2:**
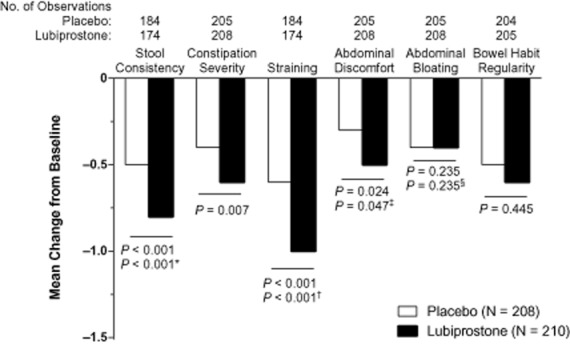
Overall change from baseline in constipation-associated symptoms in patients treated with lubiprostone 24 mcg twice daily compared with placebo (intent-to-treat population). *P* values are from the van Elteren test. Hommel's stagewise rejective method was used to adjust *P* values for the following: *stool consistency, for straining; ^†^straining, for stool consistency; ^‡^abdominal discomfort, for abdominal bloating; and ^§^abdominal bloating, for abdominal discomfort. The scale ranged from 0 (very loose) to 4 (very hard; little balls) for stool consistency, from 0 (absent) to 4 (very severe) for severity of associated symptoms, and from 1 (very regular) to 7 (very irregular) for bowel habit regularity. No imputation of missing data was performed.

**Figure 3:**
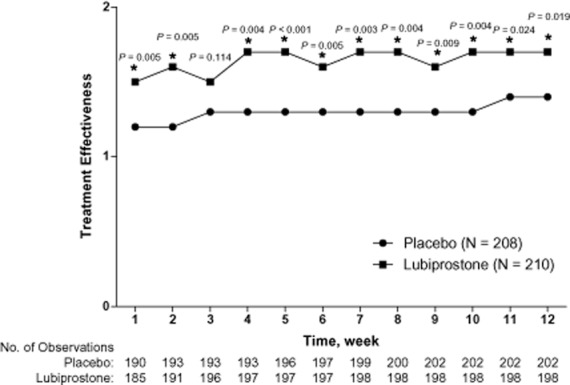
Overall effectiveness of lubiprostone 24 mcg twice daily compared with placebo in the intent-to-treat population. *P < 0.05 by van Elteren test stratified by pooled site. Missing data were imputed using the last observation carried forward (LOCF) method. Results from non-LOCF analysis were similar at almost all treatment weeks. The scale ranged from 0 (not at all effective) to 4 (extremely effective).

Patients treated with lubiprostone compared with placebo showed overall improvement from baseline in most constipation-associated signs and symptoms (Figure 2) based on patient self-assessments recorded in diary entries. Thus, pairwise comparisons showed improvements that significantly favored lubiprostone over placebo for abdominal discomfort (*P* = 0.024), straining (*P* < 0.001), constipation severity (*P* = 0.007), and stool consistency (*P* < 0.001). Patients reported, on average, a change in stool consistency from hard at baseline to approximately normal after lubiprostone treatment. Lubiprostone also resulted in an overall change in straining from moderate-to-severe at baseline to mild-to-moderate. Abdominal bloating and bowel habit regularity were not significantly improved with lubiprostone compared with placebo; however, there was a slightly larger improvement from baseline in the bowel habit regularity score with lubiprostone (−0.6) compared with placebo (−0.5). Patient ratings of overall treatment effectiveness were significantly better for lubiprostone compared with placebo at all postbaseline time points (Figure 3; *P* < 0.001 to *P* = 0.024) except week 3.

### Use of Rescue Medication

Rescue medications such as bisacodyl suppositories and enemas were permitted in certain situations during double-blind treatment at the discretion of the investigator. The use of any kind of rescue medication was similar in the lubiprostone and placebo arms at month 1 (100/207 [48.3%] vs 100/206 [48.5%], respectively; *P* = 0.962); there was a nonsignificant trend for a reduced use of rescue medications in patients treated with lubiprostrone as compared with patients receiving placebo at months 2 (63/175 [36.0%] vs 78/184 [42.4%]; *P* = 0.216) and 3 (49/155 [31.6%] vs 67/160 [41.9%]; *P* = 0.059). Among all patients, suppositories were the most commonly used rescue medication (approximately 41% of patients), followed by enemas and other treatments in approximately 22% and 26% of patients, respectively.

### Safety

Overall, 244 of 414 patients (58.9%) who received ≥1 dose of study medication experienced at least 1 AE, including 112 of 206 patients (54.4%) randomized to placebo and 132 of 208 (63.5%) randomized to lubiprostone (Table 2). Nausea, diarrhea, and abdominal distention were the most frequently reported AEs; there was a significantly higher incidence of these AEs in the lubiprostone arm compared with the placebo arm. Most patients in the lubiprostone (90.9%) and placebo (93.2%) groups experienced no severe AEs. The incidence of treatment-related AEs was greater in the lubiprostone group (36.5%) than in the placebo group (23.3%). Nausea, diarrhea, and abdominal distention were the most commonly reported treatment-related AEs; each occurred with significantly greater frequency in patients randomized to lubiprostone compared with placebo (Table 2).

**Table 2:** Adverse events

AE, No. (%) of Patients	Placebo BID (N = 206)	Lubiprostone 24 mcg BID (N = 208)	*P* value*
At least 1 AE	112 (54.4)	132 (63.5)	0.072
AEs in ≥5% of either treatment arm			
Nausea	12 (5.8)	35 (16.8)	<0.001
Diarrhea	6 (2.9)	20 (9.6)	0.007
Abdominal distention	5 (2.4)	17 (8.2)	0.014
At least 1 treatment-related AE	48 (23.3)	76 (36.5)	0.004
Treatment-related AEs in ≥2% of either treatment arm			
Nausea	11 (5.3)	32 (15.4)	0.001
Abdominal distention	5 (2.4)	16 (7.7)	0.023
Diarrhea	3 (1.5)	15 (7.2)	0.006
Flatulence	5 (2.4)	8 (3.8)	0.575
Vomiting	4 (1.9)	5 (2.4)	1.000
Upper abdominal pain	7 (3.4)	1 (0.5)	0.037
Abdominal pain	1 (0.5)	8 (3.8)	0.037
Headache	4 (1.9)	7 (3.4)	0.543
Gamma-glutamyltransferase increased	5 (2.4)	2 (1.0)	0.283

AE = adverse event; BID = twice daily.

* *P* values are from Fisher's exact test.

The incidence of SAEs was similar (*P* = 0.493; Fisher's exact test) in patients randomized to lubiprostone (12/208; 5.8%) compared with placebo (8/206; 3.9%). One patient in the placebo arm had a treatment-related SAE, but no treatment-related SAEs occurred in the lubiprostone arm. There were no deaths during the study. The AEs that most commonly led to treatment discontinuation in the lubiprostone and placebo groups were nausea (4.8% vs 0%, respectively), diarrhea (1.4% vs 0%), abdominal distention (1.0% vs 0%), constipation (1.0% vs 0%), vomiting (1.0% vs 0%), and headache (1.9% vs 0.5%). Patients in both treatment arms exhibited similar (*P* ≥ 0.231) mean increases (i.e., improvements) from baseline in MFLIE nausea subscale scores during double-blind treatment; changes in the lubiprostone and placebo arms were 1.6 and 2.8, respectively, at month 1; 4.0 and 4.3 at month 2; and 4.8 and 5.3 at month 3. Lubiprostone did not interfere with opioid-induced analgesia; mean changes from baseline in BPI-SF scores for patient self-assessed pain interference, pain severity, and worst pain were similar (*P* ≥ 0.182) in the placebo and lubiprostone arms at all time points, with the exception that pain interference was significantly less at month 2 in the lubiprostone group (*P* = 0.049). Physical examinations, electrocardiogram measurements, and clinical laboratory findings were unremarkable.

## Discussion

In this randomized, double-blind, placebo-controlled study, lubiprostone 24 mcg BID significantly improved SBM frequency at week 8 and overall compared with placebo in patients with OIC and chronic noncancer pain. Importantly, improvement in SBM frequency was rapid, with the first SBM occurring within 24 hours of the first dose of lubiprostone in approximately 40% of patients and within 48 hours in more than 60% of patients. Several secondary symptomatic endpoints, including stool consistency, constipation severity, straining, and abdominal discomfort, were also significantly improved. Results of the current study were consistent with the efficacy of lubiprostone for the relief of constipation-related symptoms in patients with CIC [14],[15]. Administration of lubiprostone in patients with OIC was generally well tolerated, and the AE profile was consistent with previous reports in patients with CIC [14],[16]. However, in the present study, nausea occurred in 16.8% of patients with OIC treated with lubiprostone 24 mcg BID compared with 21.0% to 31.7% of patients with CIC treated with the same dose in phase III studies [14],[15]. Variations in the incidence of nausea across different disease conditions could reflect different sample sizes or population characteristics, or greater emphasis in recent studies on taking doses with food. Lubiprostone did not interfere with the analgesic effect of opioids, but rather selectively addressed OIC. Patients in this study used a large mean dose of opioids (in excess of 250 mg/day of morphine equivalents) compared with the usual maximum dose for initial titration (180 mg/day) [17], suggesting that lubiprostone may improve SBM frequency and other gastrointestinal parameters in the typical population of patients with OIC.

Efficacy, tolerability, and convenience are all important factors for managing OIC in patients with chronic noncancer pain [18]. Currently, no medications other than lubiprostone are indicated for OIC in patients with chronic noncancer pain. Methylnaltrexone, a peripherally restricted opioid antagonist [7] administered via subcutaneous injection, is approved to treat OIC in patients with advanced disease in palliative care for no more than 4 months [19]. Naloxone is orally administered but is not approved to treat OIC and has a narrow therapeutic index [18]; naloxone added to oxycodone in a prolonged-release formulation has been shown to provide effective analgesia while reducing OIC-related adverse effects [20] but is unavailable in the United States. Alvimopan is orally administered and has shown some promise for treating chronic OIC 21–24, but it is currently approved only for short-term use to treat postoperative ileus [25].

The present study may be limited by examining only the 24 mcg BID dose of lubiprostone. This dose was determined to be optimal for treating patients with CIC [16] whose baseline constipation severity was more similar to that reported by patients with OIC than IBS-C. Thus, it is likely that the 24 mcg BID dose was necessary to achieve efficacy in many patients with OIC. However, when patients were analyzed regardless of dose reduction (e.g., due to severe nausea or diarrhea), results for the primary efficacy endpoint were nearly unchanged. This finding suggests that, for patients who experienced nausea or diarrhea with the 24 mcg BID dose of lubiprostone, there was still evidence of efficacy at the reduced dose. Change from baseline in SBM frequency was chosen as the primary efficacy endpoint for this study because this was the usual practice when the study was designed; however, currently, SBM response rate would be the preferred primary efficacy endpoint, based on FDA guidance. Statistical significance was not reached for some secondary endpoints, perhaps due to the small sample sizes that were chosen to power analysis of the primary endpoint. The absence of statistical significance between the lubiprostone and placebo groups in SBM frequency at 12 weeks also could be interpreted as a tolerance effect, as occurs with prolonged use of laxative-purgative agents. However, data from a 36-week extension study in this patient population (to be published separately), in which all participants received open-label lubiprostone, suggest that increases from baseline in SBM frequency are maintained long term, although this conclusion is tentative because of the lack of a comparator treatment [26]. A large number of sites contributed patients to the present study, resulting in small samples from some locations; although this could have introduced some variability, it probably also ensured that the findings are representative of a broad cross-section of health care facilities. The results of this study in patients with chronic noncancer pain may not be applicable to patients with cancer-related pain. An area for future research could be to develop as-needed dosing of lubiprostone, which may better accommodate variations in the onset and severity of OIC symptoms.

In conclusion, lubiprostone is an orally administered agent that effectively relieves many of the constipation-associated symptoms of OIC and is well tolerated in patients with chronic noncancer pain. Because lubiprostone acts to directly stimulate chloride and fluid secretion without affecting opioid-induced central analgesia, it offers a unique treatment option for patients with OIC.

## Supporting Information

Fig S1 Patient disposition.
